# Influence of the Specific Surface Area of Graphene Nanoplatelets on the Capacity of Lithium-Ion Batteries

**DOI:** 10.3389/fchem.2022.807980

**Published:** 2022-02-04

**Authors:** Iván Esteve-Adell, María Porcel-Valenzuela, Leire Zubizarreta, Mayte Gil-Agustí, Marta García-Pellicer, Alfredo Quijano-Lopez

**Affiliations:** ^1^ Instituto Tecnológico de La Energía, Avenida Juan de La Cierva, Paterna, Spain; ^2^ Instituto de Tecnología Eléctrica, Universitat Politècnica de València, Valencia, Spain

**Keywords:** anode, energy storage, graphene nanoplatelets, lithium-ion batteries, surface area

## Abstract

In order to understand the influence of the morphological properties of graphene materials on the electrochemical performance of electrodes for lithium-ion batteries, three different graphene nanoplatelets with the increasing specific surface area (NP1: 296 m^2^ g^−1^, NP2: 470 m^2^ g^−1^, and NP3: 714 m^2^ g^−1^) were added in the electrode formulation in different ratios. Higher specific surface area graphene nanoplatelets (NP3) exhibit reversible capacity up to 505 mA h g^−1^ in the first discharge cycle (29.5% higher than that of graphite). Although significant irreversible capacity is shown for NP3, still higher reversible capacity is obtained compared to that of graphite electrode. The presence of micropores in the graphene structure benefits the lithiation. C-rate capability tests also show better performance of the graphene-based electrode. In this work, we demonstrate that graphene nanoplatelets with high specific surface area (714 m^2^ g^−1^) improve the electrochemical performance of Li-ion battery electrodes. The relationship between specific surface area, the presence of defects, and porosity is discussed.

## Introduction

The reality of climate change has accelerated the energy transition to a more electrified society. The rapid expansion of the electric vehicle and the increased use of renewable energy sources require the development of energy storage systems with enhanced capacity. Lithium-ion batteries (LIBs) are the most used energy storage technology for portable electronic devices and are also being considered for stationary and transport applications due to their good performance, their relatively high energy density, and the significantly decrease of the costs over the last years (Diouf and Pode 2015; Schmidt et al., 2019). However, commercial graphite-based anodes used nowadays for LIBs are reaching the theoretical capacity limit (372 mA h g^−1^), which cannot satisfy the ever-increasing energy density requirements (Wu et al., 2020). The use and development of novel materials are crucial to satisfying the growing energetic society demand.

Due to its outstanding properties, such as high electrical and thermal conductivity and good mechanical strength, graphene will play an important role in the development of new generation of batteries. Since its first isolation in 2004 by Novoselov et al., (2004) numerous works studying its properties have been published (Balandin 2011). Graphene can be useful in many different application fields. Energy storage is one of the research areas of greatest interest. Extensive research has been carried out in relation to graphene application in electrochemical energy storage systems. Graphene can improve the performance of electrochemical energy storage systems increasing the capability, cyclability, and security (Raccichini et al., 2015; Li et al., 2019; Dai et al., 2019).

Recent studies demonstrate the benefits of using graphene in anode formulation for Li-ion batteries as it can act as active on a non-active material. 3D graphene materials with diverse architectures [graphene balls (Wang et al., 2010), fibers (Luo et al., 2011), tubes (Dong et al., 2012), etc.] are extensively used during the past years for energy storage applications since they inherit the outstanding properties of 2D graphene sheets (Chabot et al., 2014; Li et al., 2019). The advantages such as a porous network interconnected with high electrical conductivity and good mechanical properties like flexibility make this 3D architecture suitable to fabricate composites. 3D structures allow for the fabrication of graphene-based electrodes with metal-host in the porous structure decreasing the aggregation effect, enhancing the electron transfer, facilitating the diffusion of the electrolyte, and mitigating the volume expansion of the loaded metal-host. Many different materials such as alloys, metal oxides, and metal sulfides have been used in the fabrication of 3D graphene composites as anodes for LIBs (Wang et al., 2014; Zhu et al., 2016; Ren et al., 2016; Lee et al., 2017; Yang et al., 2020; Zhang et al., 2021). Although benefits are shown, these materials still need to be improved for commercial applications. To obtain these 3D graphene architectures, chemical vapor deposition (CVD) and self-assembly methods are used, requiring high cost and multi-step experimental procedures. Additional steps are necessary for metal encapsulation and composite fabrication. Moreover, these techniques are not ready for scalability to industrial applications. Thus, the use of less complex graphene-based materials, with simple synthetic procedures, easy to be incorporated in the electrode formulation and facile to scale up is mandatory to be implemented to industries.

Ternary Sn–Sb–Cu alloy nanoparticles with a specific hollow structure and uniform particle size of 10–20 nm have been described by Yang et al. as an anode material for lithium-ion batteries. A relatively high electrochemical capacity (380 mAh g^−1^) after 30 cycles with 82.6% capacity retention was obtained (Yang et al., 2020). During electrochemical reactions, the inactive copper phase serves as a soft and ductile matrix, alleviating the severe volume change during cycling. Recent work reported a novel anode material consisting in encapsulated Sn(OH)_4_ nanoparticles into the micropores of MCMBs (mesocarbon microbeads) (Zhang et al., 2021). The electrochemical results demonstrate that the Sn(OH)_4_/MCMB anode exhibits a high reversible capacity of 904 mAh g^−1^ after 500 cycles at a current density of 100 mA g^−1^. The extraordinary reversible capacity obtained with this material is attributed to the ultrasmall particle size of Sn(OH)_4_ inside the micropores, producing a decrease of the SEI formation, accommodation of electrode expansion, and a stable cycling performance due to the encapsulation. With these results, the benefits of the use of hybrid materials for anode preparation are demonstrated.

Two-dimensional graphene-based materials such as graphene nanoplatelets (GNPs), graphene nanosheets, or reduced graphene oxide (rGO) have also been studied. A series of graphene nanosheets obtained from both thermal exfoliation and wet chemical reduction in different reducing agents were studied by Vargas C. et al. (2012). For the thermal exfoliation graphene sheets, a correlation was found between the O-based functional groups. The reversible capacity increases with a decrease of the C/O ratio. However, the performance of graphene nanosheets for LIBs could not be clearly predicted after the electrochemical properties of the different graphene materials were studied.

Some studies reported the lithium adsorption enhancement in the presence of oxygen groups or surface defects exhibiting a capacity even exceeding a graphene theoretical value of 744 mA h g^−1^ (Dahn et al., 1995; Uthaisar et al., 2009; Farjana et al., 2017a). The presence of defects or edges in graphenic structure produces higher charge transfer from Li-ion to graphene. Porosity is also an important parameter that may offer some advantages to Li insertion allowing better accessibility for the electrolyte toward graphene layers enhancing the specific capacity (Farjana et al., 2020).

Also, the importance of correlation between preparation methods, structural features, and electrochemical lithium storage behavior of reduced graphene oxide has been studied by Farjana et al. (2017b). Differences in the morphology and structure of graphene materials are expected to have significant impact on the properties, thus leading to a poor understanding and prediction of their electrochemical storage in LIBs. Further studies evidence the important role of defective/disordered graphene nanosheets in the enhancement of the reversible capacity of LIBs (Pan et al., 2009).

With these antecedents, it is of imperative importance to predict the influence of physicochemical, morphological, and textural properties of graphene on the electrochemical performance of electrodes for LIBs. As is well known, there are many different types of graphene-based materials, with differences in their characteristics between each other affecting the intrinsic properties of the material. The prediction of the performance of a particular graphene material is still the key for the development of improved graphene-based electrodes for Li-ion batteries.

In the present work, the influence of the specific surface area on the electrochemical performance of electrodes for LIBs was studied. Three graphene nanoplatelets with the same morphological and physicochemical properties only varying the specific surface area were evaluated. Moreover, a facile experimental procedure to prepare graphene-based electrodes is used. Graphene nanoplatelets are incorporated as an additive in the electrode formulation, following the typical industrial procedure being easy to scale up.

## Experimental

### Materials

Three commercial graphene nanoplatelets (GNPs) from Nanografi Nano Technology are used to evaluate the influence of the specific surface area on the electrochemical performance of electrodes for lithium-ion batteries. GNPs have different specific surface areas and a similar layer lateral size and composition. GNPs were used as an additive in the electrode formulation together with graphite (MTI, EQ-Lib-CMSG, D50: 19–23) and carbon black Super C65 (Timcal). All materials were used as received.

### Characterization

Transmission electron microscopy (TEM) (JEOL JEM-1010) was used to examine the size and morphology of graphene nanoplatelet layers. Raman spectra were obtained from JASCO NRS-5100 applying 532 nm laser light to determine the presence of defects, and elemental analysis (LECO CHNS 932) was carried out to analyze the material composition. Thermogravimetric measurements (Mettler Toledo TGA 2) were performed between 0 and 900°C at a heating rate of 10 K min^−1^ by flowing air, to determine graphene impurities and the oxidation temperature of graphene nanoplatelets. Specific surface areas were determined with Micromeritics ASAP 2020 using N_2_ gas as the adsorbate.

### Preparation of Electrodes

The electrode was prepared by first making a slurry by mixing the active materials (graphene nanoplatelets and graphite) with conductive carbon black Super C65 (Timcal) in a weight proportion of 94.5:1. When graphene-based electrodes were prepared, the corresponding wt% was added to the slurry formulation and the same amount of graphite was taken. For full graphite electrodes, 94.5% by weight was used. A mixture of carboxymethyl cellulose and styrene-butadiene rubber (CMC-SBR) dissolved in water was used as a ligand in a weight proportion of 4.5% to maintain the active materials fixed. The obtained slurry was used for coating the electrodes on a Cu foil of 12 µm (MTI) using a “doctor blade” technique. Further annealing for 30 min at 60°C and 30 min at 110°C was carried out.

### Electrochemical Measurements

The cells were assembled in a high-purity argon-filled glove box (H_2_O < 0.2 ppm and O_2_ < 0.2 ppm, Mbraun). The diameter of electrodes and separator is 18 mm. The half cells were assembled with a glass fiber separator (Whatman GF) impregnated by electrolyte solution 1M LiPF_6_ in a 1:1 (v:v) mixture of ethylene carbonate (EC) and dimethyl carbonate (DMC) (Solvionic). A disc of lithium metal was used as a counter electrode (99.9% Goodfellow). Electrochemical characterization was performed using an eight-channel battery tester from Neware (BTS4000). The cells were galvanostatically charged/discharged at various C-rates between the cut-off voltages of 0.003 and 2.1 V. All the electrochemical tests were performed at room temperature. The mass loading of the active materials was taken into account to calculate the capacities.

## Results and Discussion

### Graphene Nanoplatelet Characterization

**FIGURE 1:**
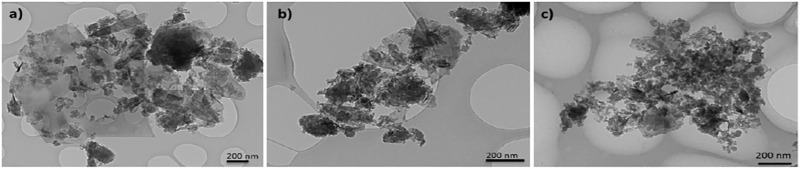
TEM images of **(A)** NP1, **(B)** NP2, and **(C)** NP3, showing their morphology.

Graphene nanoplatelet layer lateral size and morphology were studied by TEM. As shown in Figure 1, three graphene materials showed similar layer morphological characteristics. All materials have a layered structure distributed in few-layers graphene nanoplatelet aggregates, which is typical from an expandable graphite precursor. The presence of high amount of edges and defects can be seen due to the aggregate morphology and small size layers. Micron-size calculated layer lateral dimensions are determined by measuring the large amount of specimens. An average layer lateral size of around 1.3 µm was calculated for the three GNPs studied. These results indicate the morphological similarity shown between the graphene materials.

**FIGURE 2:**
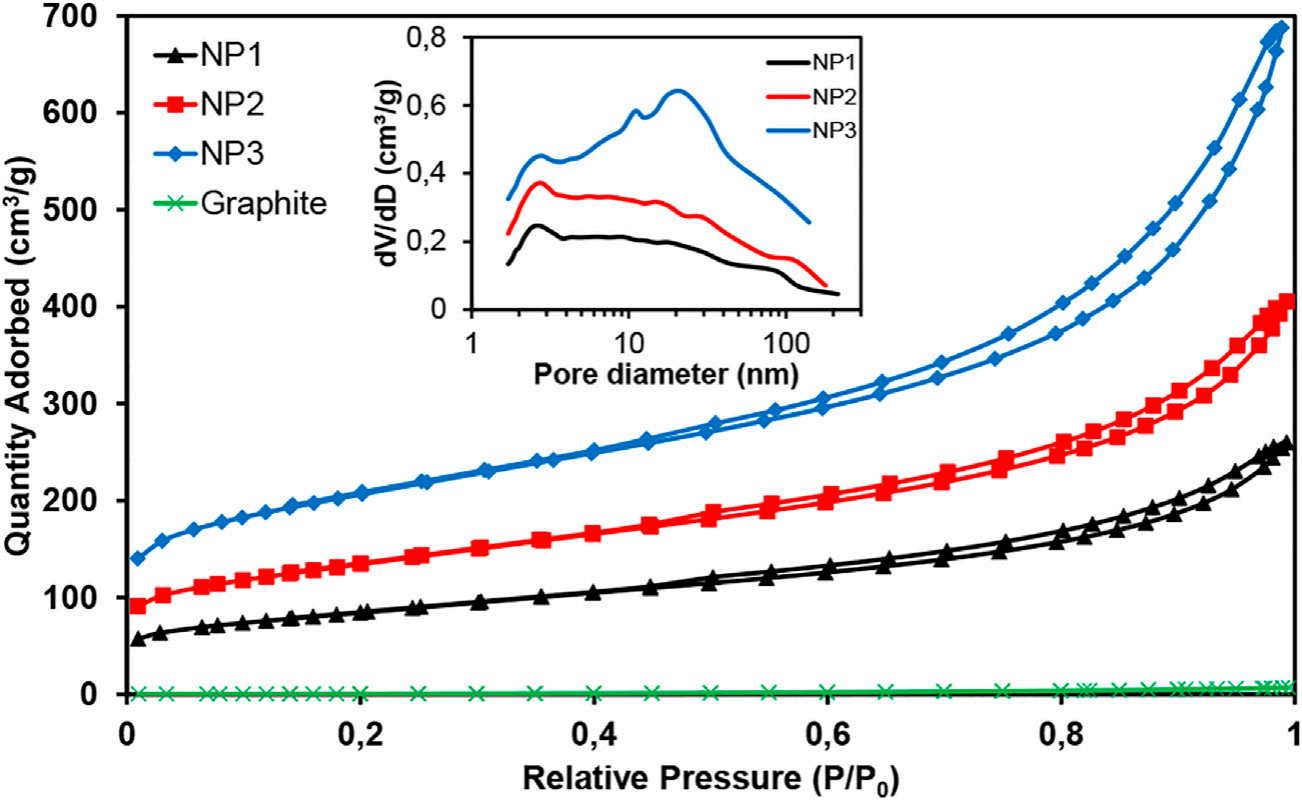
Nitrogen adsorption–desorption isotherms of graphite and three GNPs studied. Inset: pore size distribution for GNPs.

Main differences for GNPs used in this study are found in their specific surface area. Nitrogen adsorption–desorption isotherms obtained (Figure 2) show clearly different gas adsorbed quantity for NP1, NP2, and NP3. Micro- and mesoporosity are present in the graphene materials extracted from the isotherm type behavior (types I–IV). A typical hysteresis cycle can be observed in the desorption process indicating the presence of mesoporosity in the graphene structure. A BET specific surface area of 296, 470, and 714 m^2^ g^−1^ for NP1, NP2, and NP3, respectively, was calculated. Pore volume shows a small amount of micropores increasing when the specific surface is higher: NP3 > NP2 > NP1 (Table 1). A similar pore size distribution is observed for NP1 and NP2; however, substantial differences in pore size distribution are observed for NP3 (Figure 2). The highest surface area GNP (NP3) has many more mesopores (2–50 nm) and macropores (>50 nm) compared to NP1 and NP2 which leads to a bigger average pore diameter of the mesopores in NP3 than in NP1 or NP2. The reason can be attributed to a bigger interlayer space present in NP3.

**TABLE 1:** Graphene nanoplatelets’ morphological characterization.

GNPs	Isotherm type (IUPAC)	BET method	t-Plot method			I_D_/I_G_	NP layer lateral size (µm)
		S_BET_ (m^2^ g^-1^)	V_mic_ (cm^3^ g^-1^)	S_mic_ (m^2^ g^-1^)	S_ext_ (m^2^ g^-1^)		
NP1	Types I–IV	296 ± 1	0.03	59.5	236.5	0.53	1.2
NP2	Types I–IV	470 ± 2	0.05	98.9	371.5	0.63	1.3
NP3	Types I–IV	714 ± 6	0.09	185.7	528.4	0.81	1.3
Graphite	Type III	2.3 ± 0.1	0.00	0.6	1.8	0.28	D50: 19–21

**FIGURE 3:**
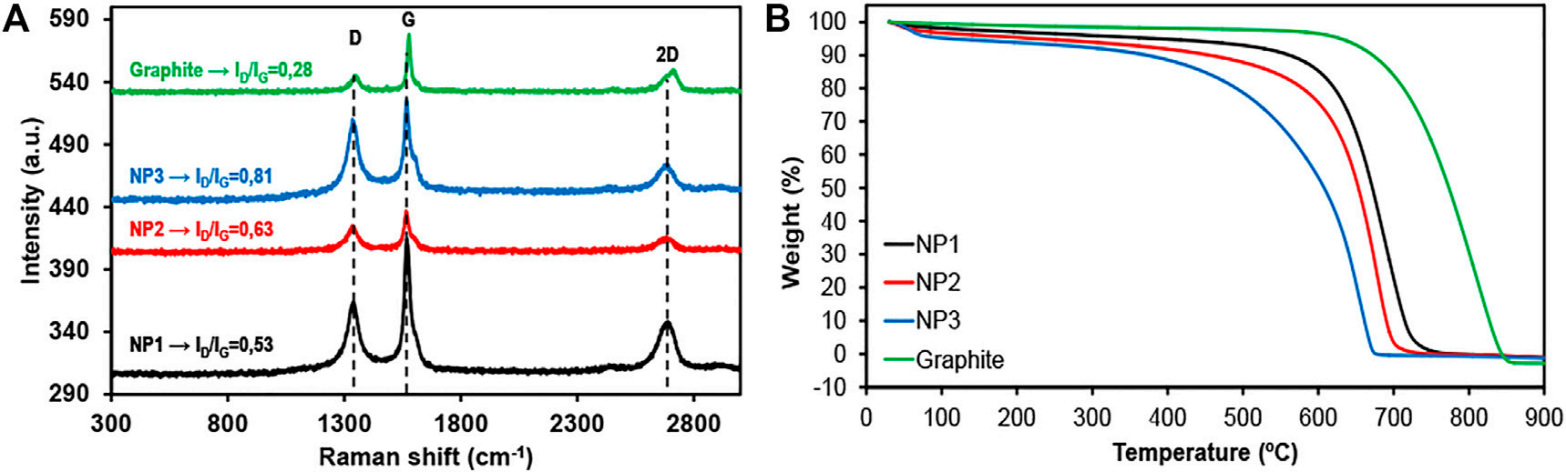
**(A)** Raman spectra of graphite and three graphene nanoplatelets recorded using a 514 nm excitation laser. The spectra exhibit the 2D, G, and D bands at about 2,750, 1,590, and 1,350 cm^−1^, respectively. **(B)** TGA decomposition profile in air at 10 K min^−1^.

Figure 3A shows the Raman spectra for NP1, NP2, and NP3. Typical graphene bands, namely, G and 2D, appear at 1,590 and 2750 cm^−1^, respectively. Additionally, a third band appears at 1350 cm^−1^ corresponding to the D band associated with the defects in graphene layers, including both inner and surface defects. This band can be attributed to the presence of heteroatoms, defects, or edges in the graphene layer structure. Due to the small lateral size observed for these materials, the D band is associated mainly with the presence of large amount of sheet edges where carbon vacancies and dangling bounds are present. An increase in the I_D_/I_G_ value indicates more disordered carbon present in the graphene layer structure. Sheet boundaries caused by the high surface area of GNPs studied have an important contribution to the D band. As can be seen in Figure 3A, the I_D_/I_G_ ratio is closer to 1 for the highest specific surface area GNP. The ratio between the intensity of D and G bands is 0.53 for the lower surface area GNP (NP1, 296 m^2^ g^−1^) and increases to 0.63 for NP2 with 470 m^2^ g^−1^ and 0.81 for the highest surface area graphene (NP3, 714 m^2^ g^−1^). The increase of I_D_/I_G_ ratio is also associated with the presence of oxygen in the structure. Table 2 shows an increase in the oxygen content together with specific surface area from 5 to 13 wt%. The high I_D_/I_G_ ratio observed for NP3 together with the highest specific surface area and bigger pore size distribution compared to NP1 and NP2 indicates the presence of more defective and disordered carbonaceous structure which is beneficial to improving the lithium storage capacity (Xiang et al., 2012). Graphite Raman spectra are also shown in Figure 3A with the characteristics G and 2D bands, together with a low intensity D band. As reported in bibliography graphite, the 2D band is shifted to the right with respect to graphene (Roscher et al., 2019).

**TABLE 2:** Graphene nanoplatelet elemental analysis.

GNPs	C (wt%)	H (wt%)	N (wt%)	S (wt%)	O (wt%)
Graphite	98.831	0.000	0.000	0.000	1.169
NP1	94.144	0.000	0.146	0.000	5.710
NP2	91.552	0.000	0.168	0.014	8.266
NP3	86.262	0.003	0.430	0.098	13.207

Thermal analyses were carried out to determine the presence of impurities and stability of graphene materials. Thermogravimetric analysis (Figure 3B) shows similar decomposition profiles for the three GNPs in air at 10 K min^−1^, with one mass loss step. No presence of solid residue at 900°C indicates high-purity material. Differences in the initial decomposition temperature observed for the GNPs in the TGA profile are caused mainly by the variation of the specific surface area of the materials. The greater the specific surface area, the greater the number of edges and defects existing in the structure of the material (Figure 3A), making it less stable against temperature. Therefore, as the specific surface area increases, the decomposition temperature of GNPs decreases. Weight loss differences are also in accordance with the presence of defects in the graphene structure, as can be seen in the Raman spectra in Figure 3A. Oxygen content also plays an important role in the thermal stability of graphene materials. As shown in Table 2, the oxygen content increases for the materials having more surface area, indicating the presence of higher amount of oxygenated functional groups in the structure. These functional groups are less stable against temperature; thus, the graphene materials with high oxygen content start to decompose at lower temperatures. Looking more in detail, NP3, with the highest surface area, has the smallest decomposition temperature (≈450°C) due to the presence of large amount of sheet edges and boundaries which decreases the thermal stability and due to the higher oxygen content present in the structure compared with the other GNPs. NP1 and NP2 start to decompose at 600 and 550°C, respectively, according to the lower number of defects and oxygenated functional groups in their structures. Despite this, the GNPs studied have high thermal stability. A summary of GNPs’ physicochemical and morphological characterization is shown in Table 1.

The characterization of this series of GNPs shows the same morphological and physicochemical properties only varying the specific surface area and consequently the ID/IG ratio attributed to the presence of disordered and defective carbon atoms coming from the inner porosity of the GNPs studied. The influence of the specific surface area on the electrochemical performance of graphene-based electrodes for Li-ion batteries will be studied.

### Electrochemical Characterization

**FIGURE 4:**
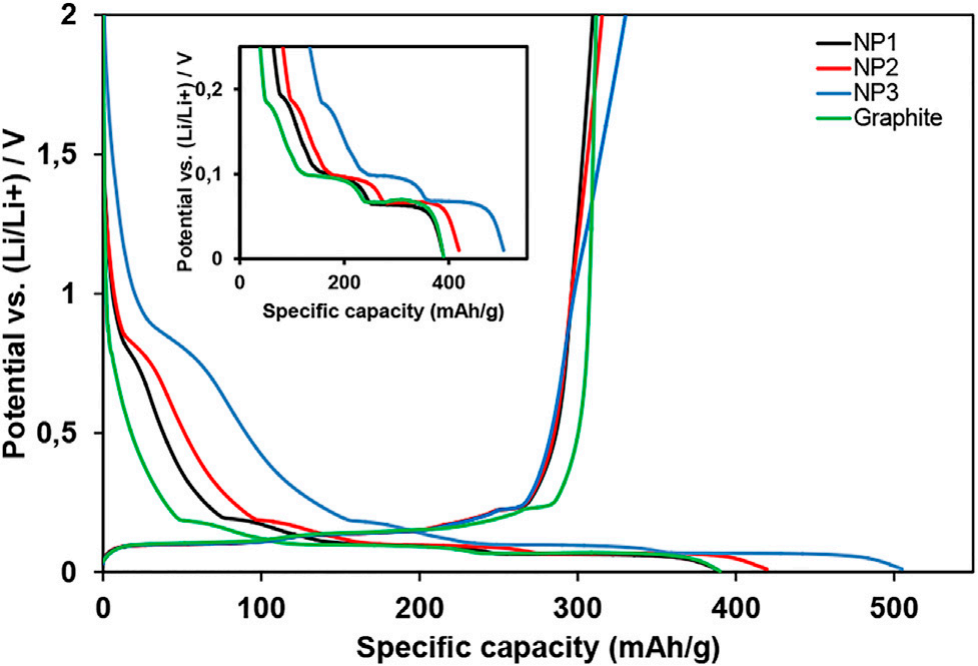
First discharge and charge curves at a current rate of C/20 for graphite and three graphene-based electrodes evaluated. Flat plateaus below ∼0.25 V (vs. Li/Li^+^) are zoomed in as an inset. Note: 10 wt% of GNPs is used in graphene-based electrodes; the total active material is 94.5 wt%.

The surface area effect on the electrochemical performance of graphene-based electrodes for LIBs was evaluated using galvanostatic charge–discharge cycling. During the first discharge curve at slow C-rates (C/20) shown in Figure 4, the presence of different plateaus below ∼0.25 V (vs. Li/Li^+^) can be observed for electrodes based on graphene nanoplatelets. All the results shown in this study correspond to electrodes containing 10 wt% of the corresponding graphene nanoplatelets in the formulation. The total active material mass loading including graphene and graphite is fixed to 94.5 wt%. The results corresponding to different graphene mass loading will be indicated explicitly. The zoomed-in discharge curves for graphite, NP1, NP2, and NP3 electrodes can be seen in the inset of Figure 4. During the first discharge, a decomposition of electrolyte occurs on the carbonaceous material surface; the products of this decomposition are called the solid electrolyte interface (SEI). The SEI is a film on the carbonaceous material which protects the electrolyte from continuous decomposition during subsequent cycling (Fong et al., 1990). The voltages plateaus that can be observed below 0.25 V (vs. Li/Li^+^) are associated with intercalation/de-intercalation processes in carbonaceous materials.

In case of graphene electrodes (NP1, NP2, and NP3), a redox process at ∼0.8 V (vs. Li/Li^+^) can be observed. It is associated with the first interlayer graphene sheet Li intercalation that occurs at a higher potential compared with plateaus placed below ∼0.25 V (vs. Li/Li^+^) (Raccichini et al., 2016).

On the contrary, an almost negligible capacity is obtained at potentials ≥1.4 V (vs. Li/Li^+^) in all materials. As reported previously by Stournara et al. using first principle calculations, the lithiation of epoxide groups takes place at those high potentials (Stournara and Shenoy 2011). Thus, no epoxide groups are present in the GNPs studied. The best performance is achieved at low oxygen content present in graphene materials (close to 12.5% oxygen), where Li can be attached to both oxygen groups (like hydroxyls) and carbon rings (forming LiC_6_). The greatest capacities are achieved for this oxygen content range with lithiation potentials close to ∼0.8 V (vs. Li/Li^+^). Due to the strength of C–O bonds, the lithiation potentials are relatively higher than the graphite lithiation potentials. The bigger interlayer space produced by disordered and defective carbon also increases the capacity in the first cycle.

GNP NP3 is the one with the highest specific surface area and thus having more edges and boundaries compared to the other materials studied (more disordered material according to the I_D_/I_G_ ratio discussed earlier). Also, taking into account the elemental analysis (Table 2), NP3 has 13% oxygen content, close to the optimal window described above. Accordingly, a higher capacity is obtained at ∼0.8 V (vs. Li/Li^+^) (Figure 4).

Capacity values obtained in the first discharge for NP1, NP2, and NP3 electrodes are 388, 420, and 505 mA h g^−1^, respectively. The lithiation capacity in the first cycle for the NP1 electrode is similar to that of the graphite electrode (390 mA h g^−1^). An increase of 29.5% for the NP3 electrode is observed indicating the good effect produced in the electrochemical performance by the high surface area present in the NP3 material.

Nevertheless, irreversible capacity loss in the first cycle, corresponding to surface reactions and SEI formation, increases also with the GNP surface area, being 17, 21, and 31% for NP1, NP2, and NP3, respectively. According to the previous observations reported (Joho et al., 2001; Bernardo et al., 2011), these results show an increase in the irreversible capacity when the specific surface area increases. The same irreversible capacity as for NP1 was obtained for the graphite electrode (17%).

**FIGURE 5:**
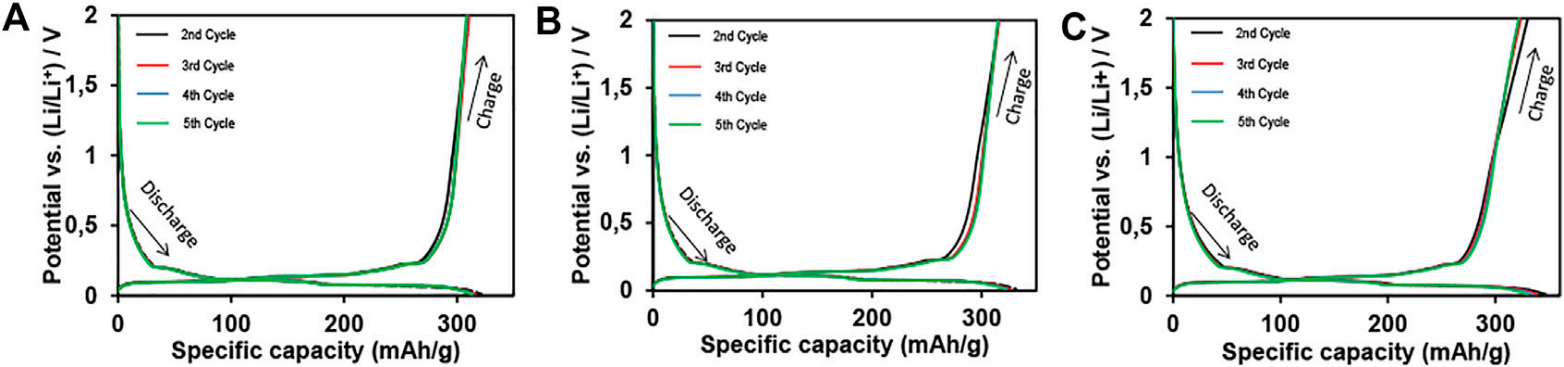
Charge–discharge profiles for the second to fifth galvanostatic cycles of **(A)** NP1, **(B)** NP2, and **(C)** NP3 electrodes at a current rate of C/20. Note: 10 wt% of GNPs is used in graphene-based electrodes; the total active material is 94.5 wt%.

In Figure 5, charge–discharge profiles for graphene-based electrodes (NP1, NP2, and NP3) are shown. A good reversible capacity can be observed, i.e., the capacity is maintained after five cycles in all GNP electrodes.

**FIGURE 6:**
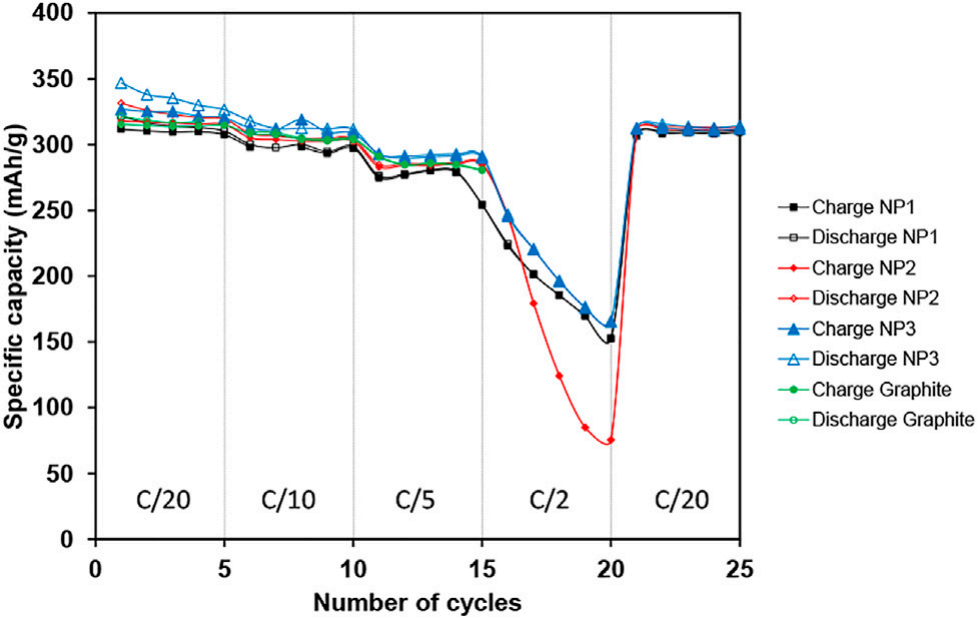
Cycling performance (charge and discharge capacity versus cycle number) of graphite, NP1, NP2, and NP3 electrodes at different C-rates. Note: 10 wt% of GNPs is used in graphene-based electrodes; the total active material is 94.5 wt%.

Figure 6 shows the cycling behavior: the charge and discharge capacity vs. the cycle number of each electrode (graphite, NP1, NP2, and NP3) at different current rates from C/20 to C/2. As can be seen, the NP1 electrode shows the lowest reversible capacity for all C-rates. At a C-rate of C/20, a reversible capacity of 312 mA h g^−1^ is obtained for the NP1 electrode in the first cycle with low fading capacity after the fifth cycle (307 mA h g^−1^). For electrodes composed of graphene materials with more specific surface area, the capacity increases. In case of the NP3 electrode, the reversible capacity obtained is 327 mA h g^−1^ in the first cycle, maintaining this value (320 mA h g^−1^) after five cycles (at C/20). The value of the specific reversible capacity for the NP2 electrode is between NP1 and NP3, 318 mA h g^−1^, retaining 315 mA h g^−1^ at C/20 after five cycles. In the case of graphite, the specific reversible Li-capacity recorded for the first cycle is 315 mA h g^−1^, and no fading capacity is observed in the fifth cycle (314 mA h g^−1^) at a current rate of C/20, as expected.

Additionally, good C-rate capability is observed for all GNPs, especially for NP3 retaining 290 mA h g^−1^ after five cycles at C/5. These can be attributed to the enhancement produced by the presence of highly conductive graphene nanoplatelets in the electrode composition. These results indicate that highly disordered carbon in the GNP structure results in a boost of the specific capacity of anodes for lithium-ion batteries. The addition of 10 wt% of huge surface area GNP in the electrode composition represents an increase in the capacity of about 4%.

**FIGURE 7:**
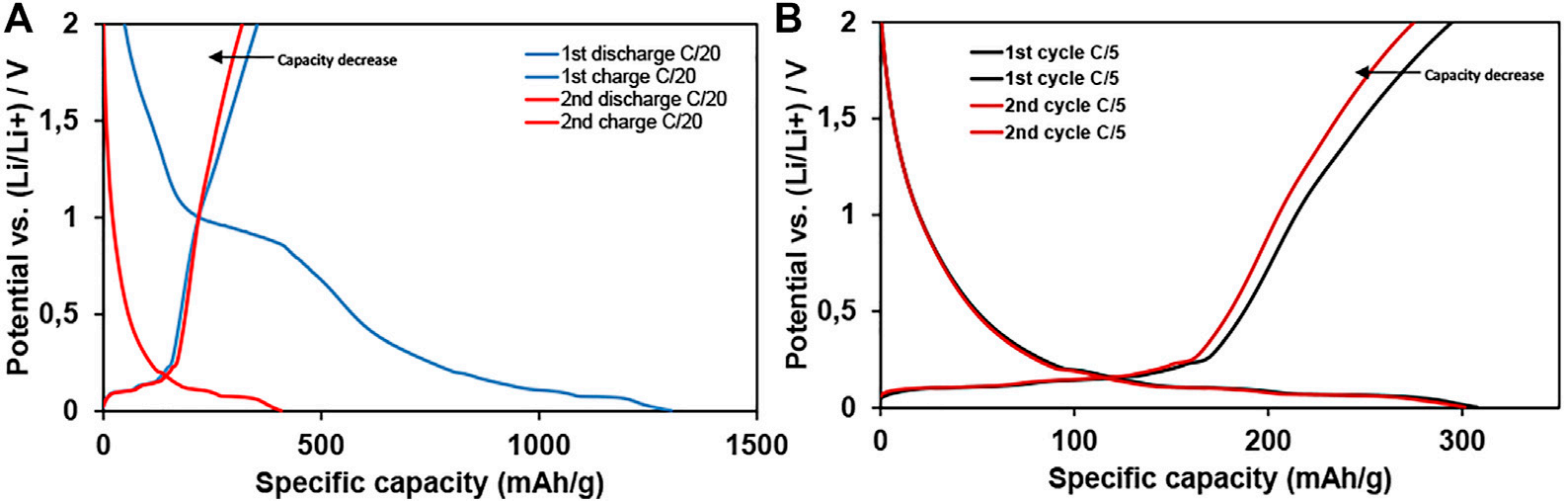
First and second charge–discharge curves for the 50 wt% graphene NP3 electrode at **(A)** C/20 and **(B)** C/5 current rates. The total active material is 94.5 wt%.

On the contrary, the increase of GNP wt% in the anode formulation was evaluated. Using 50 wt% of NP3 in the electrode composition, a huge capacity of 1305 mA h g^−1^ (at a C-rate of C/20) was achieved in the first discharge cycle indicating the influence on the lithiation capacity improvement caused by the high surface area GNP. According to the behavior observed for the 10 wt% NP3 anodes, a highly disordered graphene structure carries a significant irreversible capacity, which is 69% for the 50 wt% NP3 electrode, but still retaining up to 368 mA h g^−1^ in the first cycle after SEI formation (Figure 7). This capacity value represents an increase of ~13% compared with that of the same NP3 in 10 wt%. However, as can be seen in Figure 7A, the charge capacity reached in the second cycle was 332 mA g^−1^, a 9.8% of decrease compared to that in the first cycle. These capacity values reached with 50 wt% graphene NP3 electrodes, after the second charge–discharge, are similar to the capacity obtained with the electrodes containing 10 wt% of graphene NP3. These results show that an increase by 40% on the GNP loading in the electrode formulation does not translate to a significant increase in the reversible capacity after two charge–discharge cycles (Figure 7). C-rate capability tests also show the poor capacity of the 50 wt% graphene NP3 electrode when the C-rate increases. In Figure 7B, first and second charge–discharge curves are presented, showing a considerable decrease in the reversible capacity to 286 mA h g^−1^ after two cycles. This value is 4 mA h g^−1^ lower than the capacity obtained with the 10 wt% graphene NP3 electrodes after five cycles (Figure 5).

Thus, the best results were obtained with 10 wt% graphenic material together with graphite as active materials, obtaining stable capacities upon cycling and maintaining good capacity at different current rates. The use of high surface area GNPs as an additive in the graphite anode formulation allows tuning the electrochemical performance of the electrode. Using a certain amount of disordered GNP (in this case optimized around 10 wt%), the specific reversible capacity is improved by a factor of 4%, and it will help to go further to the theoretical graphite capacity.

**FIGURE 8:**
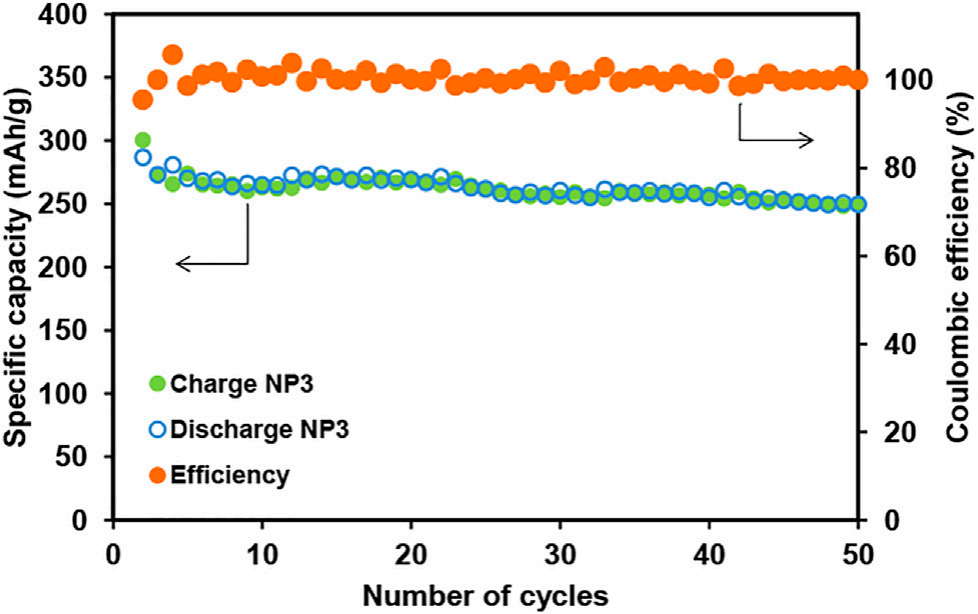
Capacity retention and coulombic efficiency for the NP3 electrode during the cycling stability test at a current rate of C/5. Note: 10 wt% of GNPs is used in graphene-based electrodes; the total active material is 94.5 wt%.

The specific capacity retention and coulombic efficiency for the NP3 electrode during the cycling stability test are shown in Figure 8. The charge/discharge capacity is maintained upon cycling at a current rate of C/5. After 50 charge/discharge cycles for the NP3 electrode, the specific reversible capacity retained is about 250 mA h g^−1^ with 100% coulombic efficiency.

The mechanism of Li insertion/disinsertion by graphenic carbon materials is still not well-established. Contradictory reports concerning lithium mechanism in graphenic materials can be found. Also, huge variation in the reversible capacity has been reported for similar graphene-based materials. This lack of consensus has been mainly related to the wide variations in the structural characteristics, such as the number and types of defects and the degree of ordering. In order to compare NP3, evaluated in this study, with other graphene-based electrodes reported in the literature, Table 3 summarizes the electrochemical behavior of previously reported graphene-based anode materials.

**TABLE 3:** Summary of the electrochemical behavior of previously reported graphene-based anode materials.

Material	Specific capacity in the first cycle (mAh/g)	First cycle coulombic efficiency	References
NP3 (this study)	505 at C/20	69%	—
GO	328 at C/20	86%	Farjana et al. (2017b)
rGO (GO reduced by hydrazine at room temperature)	400 at C/20	38%	Farjana et al. (2017b)
rGO (GO reduced by hydrazine at 80°C)	330 at C/20	46%	Pan et al. (2009)
Microwave-assisted exfoliation of GO	398 at C/10	60%	Petnikota et al. (2013)
rGO (GO reduced thermally at 500°C)	434 at C/20	51%	Xiang et al. (2012)
CVD growth on Ni foam	180 at C/5	—	Ji et al. (2019)
Electron-beam–reduced GO	1,054 at C/20	52%	Pan et al. (2009)
rGO (GO reduced thermally at 500°C with boric acid)	801 at C/10	110%	Sahoo et al. (2015)
Spindle-like CoCO3/rGO	1,473 at C/5	76%	Wang et al. (2020)

As can be seen in Table 3, many different studies have reported the benefits of graphene materials improving the electrochemical performance of anode electrodes for lithium-ion batteries. Some studies report exceptional capacity in the first charge–discharge cycle, with a significant capacity fading upon cycling. Doping and hybrid materials using metal oxides have been demonstrated as good strategies for electrode capacity improvement. Some works reported a higher specific capacity compared to the one achieved in this work. Sahoo et al. described an rGO obtained upon thermal reduction of GO at 500°C in the presence of boric acid, achieving 801 mA h g^−1^ of capacity (Sahoo et al., 2015). Boron-doped rGO increases the electrochemical performance of the electrode. Wang et al. also reported an oxide hybrid anode material based on CoCO_3_/rGO with 1,473 mA h g^−1^ of capacity at C/5 (Wang et al., 2020). The use of oxide as dopants of graphene structures improves the electrochemical performance. Nevertheless, some of these works reporting huge reversible capacity graphene-based electrodes use expensive and complex methods like CVD, tedious thermal reduction methods for material preparation, and difficult procedures for hybrid metal oxide–graphene material synthesis. These experimental procedures are difficult to scale up due to the processability of the materials. As already commented, another important point is understanding the influence of the structural features of graphene materials on the electrochemical performance. In our work, the influence of the specific surface area of graphene nanoplatelets was evaluated. Higher specific surface area graphene nanoplatelets increase the specific capacity of the anodic electrodes. Additionally, we developed and validated a simple and easy-to-scale-up method for electrode preparation following the actual industrial procedure used for graphite electrode preparation. Accordingly, our work represents a step forward to understand the benefits of the different features of graphene materials in electrochemical energy storage and to bring the use of graphene closer to the electrode-manufacturing industry.

## Conclusion

Morphological properties of graphenic materials have considerable impact on the electrochemical Li storage of graphene-based electrodes. In order to have a comprehensive understanding of how their structural and compositional features influence the electrochemical performance of electrodes, a series of graphene nanoplatelets with the increasing specific surface area (NP1: 296 m^2^ g^−1^, NP2: 470 m^2^ g^−1^, and NP3: 714 m^2^ g^−1^) were evaluated. Morphology and layer lateral size distribution evaluated by analyzing TEM images show the same distribution for all graphene materials of 1.3 µm. An increase in the surface area introduces more defects on the graphene structure (the I_D_/I_G_ ratio increases from 0.53 (for NP1) to 0.63 (for NP3) and 0.81 (for NP3)). A significant increase in the presence of microporosity on the NP3 graphene structure is also observed. The presence of large amount of extrinsic/intrinsic defects in the NP3 graphene structure and higher microporosity produce an enhancement in the reversible capacity up to 505 mA h g^−1^ in the first discharge cycle for electrodes prepared with 10 wt% of graphene, representing an improvement of 29.5% compared to that of only graphite-based electrode (390 mA h g^−1^). Nevertheless, irreversible capacity loss in the first cycle increases also with the GNP surface area, which is 17, 21, and 31% for NP1, NP2, and NP3, respectively. Although high irreversible capacity is obtained for NP3, an improvement of the reversible capacity compared to that of the graphite electrode after five cycles (at C/20) is obtained (320 mA h g^−1^). Additionally, good C-rate capability is observed for all GNPs, especially for NP3 retaining 290 mA h g^−1^ after five cycles at C/5. These results indicate that highly disordered carbon in the GNP structure results in a boost of the specific capacity of anodes for lithium-ion batteries.

From this study, we conclude that the presence of extrinsic/intrinsic defects in the graphene structure is responsible for this enhancement of the reversible capacity. By tuning the wt% of graphene in the electrode formulation, the electrochemical performance of the batteries can be modified depending on the target application. From the industrial point of view, an accurate understanding of the influence of graphene morphological properties on the electrochemical performance of electrodes for Li-ion batteries is crucial. Nowadays, graphene nanoplatelets are sold in large scale by quite a lot of manufacturers around the world. Many different types of graphene nanoplatelets with specific physicochemical and morphological properties can be chosen. Understanding the influence of each of these properties and the proportion on the electrochemical behavior of electrodes for Li-ion batteries will help to select the most adequate graphene material for a given application.

## Data Availability

The original contributions presented in the study are included in the article/Supplementary Material, and further inquiries can be directed to the corresponding author.
